# The effect of canal curvature on cyclic fatigue resistance of rotary instruments using different irrigation materials ( *in vitro* study)

**DOI:** 10.12688/f1000research.130249.2

**Published:** 2024-04-08

**Authors:** Mohammed Hamoudi Alsunboli, Sally Saad Ali Ihsan, Duha Qais Sabah

**Affiliations:** 1 College of Dentistry, Al-Bayan University, Baghdad, Iraq

**Keywords:** cyclic fatigue, rotation, fracture, M wire, autoclave, irrigation, curve canal

## Abstract

**Background:**

The mechanical qualities of Ni-Ti is crucial because they give the files their flexibility and enable us to prepare curved and double-curved canals with more ease. It happens frequently for instruments to separate during canal preparation, and cyclic fatigue (metal fatigue) is a frequent cause.

This study aimed to assess how irrigation affected the two rotary endodontic instruments’ cyclic fatigue resistance.

**Methods:**

The Edge File and Fanta File rotary endodontic instrument groups were chosen. Each group (n = 42) was split into 3 subgroups (n = 14 each), one receiving NaOH, one Glycine, and one EDTA treatment. The number of cycles to failure (NCF) was determined after each subgroup underwent testing for cyclic fatigue resistance.

**Results:**

The result appeared different significant between the two group and sub-group with the different materials that used with it with the length of fractures and time that recorded in each group.

**Conclusion:**

NaOCl, glycine, and EDTA as chemical materials appeared to have considerably varied cycle fatigue resistance for various lengths of fractures and durations, according to the comparison between the two evaluated instruments.

## Introduction

The success of endodontic therapy is associated with the cleansing and shape of the root system, which is impacted by mechanical elements such the preparation system and preparation process. The effectiveness of chemical agents with irrigation solutions in tissue solution, germination, and residue removal is covered next ( Estrela *et al.*, 2003).

Nowadays, there is increased use of the term “modern endodontics” to refer to contemporary applied science and actual materials that have been introduced and invented in recent years. Diverse endodontic tools and technological devices have been designed to facilitate and enhance treatments.

The durability of a rotary endodontic tool inside an angulated root canal is determined by the extent of flexing it experiences, influencing its longevity before succumbing to fatigue. A assessed the cyclic fatigue arises when the instrument shaft still rotates with the endodontic file’s tip, or a fragment of it, lodged within a canal. In such cases, the metal surpasses its elastic limits, resulting in plastic deformation and eventual fracture (Berutti *et al.*, 2012).

In modern endodontics, NiTi rotary devices universally shape root canals ( Torabinejad and Walton, 2009). They exhibit superior flexibility and cutting efficiency compared to standard stainless steel files ( Schafer *et al.*, 2004). The remarkable flexibility simplifies achieving the desired tapering root canal form with a reduced risk of canal transportation ( Chen and Messer, 2002). Despite these advantages, NiTi instruments are susceptible to separation ( Arens *et al.*, 2003), primarily due to wear and torsional shear forces ( Varela-Patino *et al.*, 2010; Berutti *et al.*, 2012). Cyclic fatigue occurs during rotating instrumentation in a curved canal, subjecting the instrument to repeated cycles of compression and tension ( Chen and Messer, 2002). Operational speed, metal surface treatment, and metallurgical characterization of NiTi alloys are among the variables studied for potential influences on the fatigue resistance of NiTi rotary files ( Gambarini *et al.*, 2008).

Canal curvature, attributed to flexural loads and cyclic fatigue, is considered the primary cause of instrument failure ( Hulsmann *et al.*, 2005). Corrosion in the presence of an irrigating solution is another factor that may diminish resistance to fatigue fracture ( Sonntag and Heithecker, 2006). The gold standard for tissue disintegration and disinfection involves irrigating root canals with NaOCl and EDTA ( Torabinejad and Walton, 2009). NiTi instruments, when used in root canal instrumentation, come into contact with these irrigating solutions. Consequently, corrosion patterns, involving the selective removal of nickel from the surface, may lead to micro pitting, compromising the structural integrity of the instrument. Clinicians have limited options to mitigate such stresses (Berutti *et al.*, 2012). Aim of study: This study aimed to assess how irrigation affected the two rotary endodontic instruments’ cyclic fatigue resistance in a curve canals devices.

## Methods

For this study, we utilized the Edge File (0.25/0.6, USA), distinguished by its proprietary heat-treated FireWire NiTi construction, renowned for its flexibility and durability. This nique alloy offers a blend of strength and flexibility. Additionally, we employed Fanta files (0.25/0.6, China), crafted through advanced metallurgical methods, to achieve smooth, straight-line access, augmenting flexibility and cutting efficacy. Their exceptional taper design facilitates large canal shaping.

The sample size calculation was based on the results of previous study ( Hasegawa *et al.*, 2014). Using an alpha (α) level of 0.05 (5%) and a beta (β) level of 0.20 (20%) (i.e., power = 80% at a 5% significance level) and a difference between the two groups of 50 ± 51.3, the minimum estimated sample size was 10 samples per group but we increased it to 20 samples per group ( Plotino *et al.*, 2006).

Groups of rotary endodontic instruments were carefully selected, each group consisting of three subgroups, resulting in a total of 42 samples (n = 42), with 14 samples in each subgroup.

The subgroups were designated as follows:

Subgroup 1: Treated with glycerin

Subgroup 2: Treated with EDTA 17%

Subgroup 3: Treated with sodium hypochlorite 5.25%

Considering the diverse canal shapes found in natural teeth, conducting cyclic fatigue tests consistently with natural teeth is impractical.

This study employed artificial canals constructed from fixed tapered stainless steel. These canals were placed in a water bath at 37°C, specifically designed for this research. The synthetic canals featured regular (60°) curvature angles, a 5 mm radius of curvature, and a gradual reduction in the width of the coronal portion from 1.5 mm to 1 mm at its end ( Plotino and others, 2009).

The testing followed the manufacturer-recommended settings for the rotary system (500 Rpm, 2.5 N torque) for both Fanta and Edge systems. The file tip, set to its full working length, was positioned 5-7 mm from the center of the simulated curvature (19 mm). The working area spanned 25 mm, and all files were new. The cyclic fatigue tests involved rotating the file as static rest freely inside the tapered section of the artificial canal, simulating the file’s restraint in the canal’s curved section ( Schneider, 1971).

To facilitate hand-piece movement and file insertion, the dental hand-piece was mounted on a wooden block, ensuring uniform file depth placement and three-dimensional alignment. A transparent plastic sheet prevented file slippage and allowed researchers to observe file usage and fracture development. The wooden block was secured to the stainless steel block to minimize movement ( Yılmaz *et al.*, 2017).

**Figure 1.**
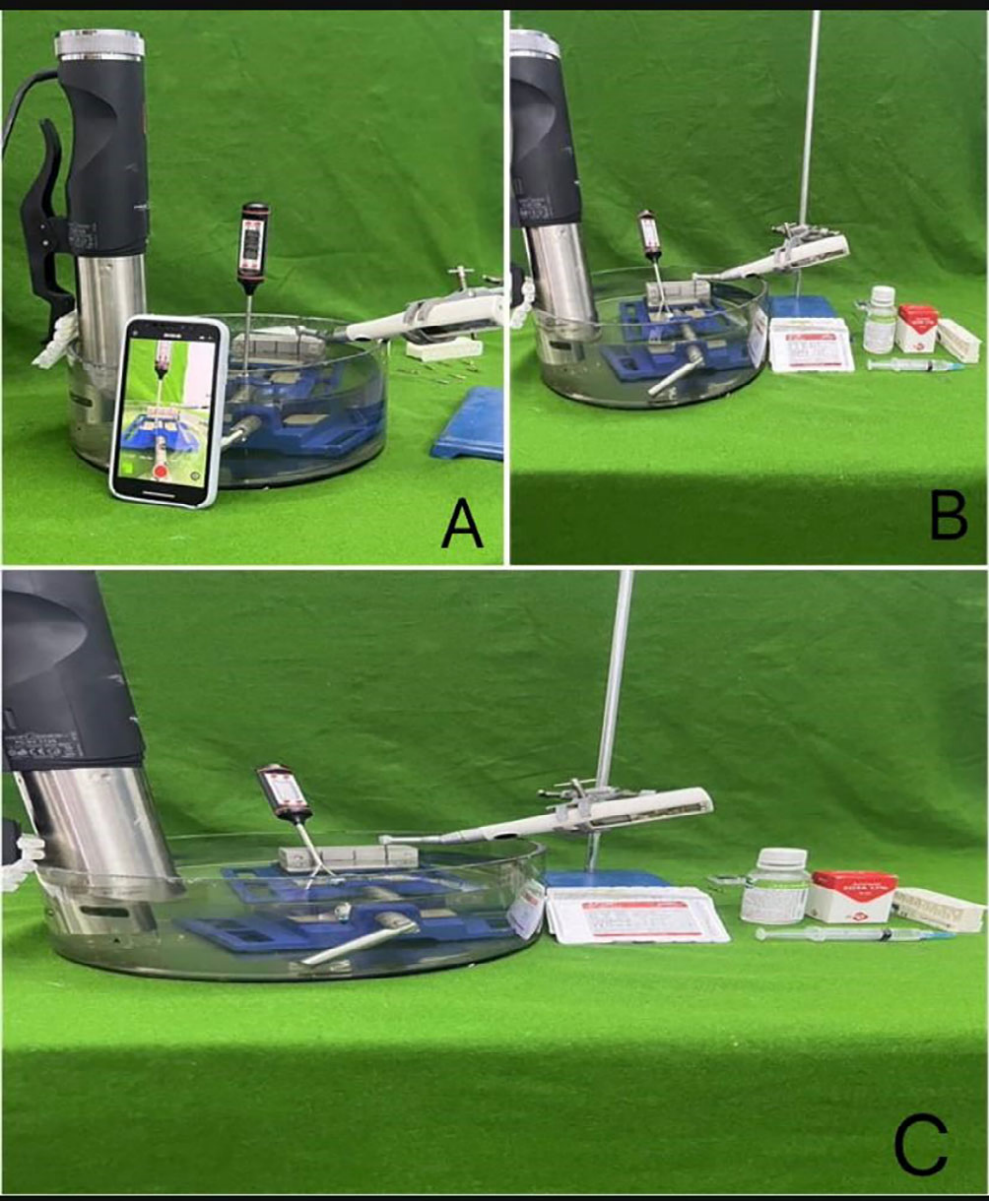
The armamentarium used in this study.

Glycerin in subgroup 1 was used to reduce heat and friction, packed inside the artificial canal before each file’s insertion. The files were activated inside the canals using the cordless endodontic hand-piece (ENDOMAX PLUS cordless endodontic handpiece) at speed 500 rpm and tourque 2.5N, with concurrent video recording t by mobile video camera (iphone 13 promax) enhance productivity and reduce errors ( Bhagabati *et al.*, 2012). The number of cycles to failure (NCF) was described by the equation: “NCF = Speed (RPM) × Time (T) to fracture in minutes.” The study’s armamentarium is depicted in Figure 1.

After being removed from the solutions, each file dried, given an ID number, and stored in glass vials.

Then, using a mechanical device created expressly for the job and capable of simulating the conditions of an instrument encased in a curved canal, instruments from all three groups and each brand were put through cyclic fatigue testing ( Grande *et al.* 2006; Plotino *et al.* 2009).

The apparatus was connected to the same dynamic immersion programs and motor set. This made it possible for the endodontic instruments to freely rotated and maintain constant pressure inside a stainless steel artificial channel. To make the artificial canal, the instrument’s dimensions and taper were reproduced. The time to fracture (TtF) for each instrument, measured in seconds from the start of the test until the point of breakage, was recorded by caliper and registered using a chronometer to the nearest whole number. The TtF was the dependent variable, whereas the type of files used and the immersion conditions were independent factors.

### Statistical analysis

The Statistical Analysis System - SAS (2018) application was used to determine the effects of various factors on the research parameters. The least significant difference (LSD) test was used in this investigation to compare the means in a significant manner (ANOVA).

## Results

Interfacial statistics of TtF for each file is summarized in Tables 1 and 2.

**Table 1.** Effect of type of File and chemical materials in length of fracture.

Chemical materials	Mean ± SE of length of fracture		LSD	P-value
	Edge file	Fanta file		
Glycerin	3.02 ± 0.17 C b	3.65 ± 0.16 A a	0.490 *	0.0136
EDTA	4.74 ± 0.11 A a	3.45 ± 0.09 A b	0.304 **	0.0001
Sodium hypo-chloride	3.73 ± 0.12 B a	3.28 ± 0.16 A b	0.421 *	0.0371
LSD	0.397 **	0.413 NS	---	
P-value	**0.0001**	**0.199**		

Means with different big letters in the same column and small letters in the same row are significantly different.

* P ≤ 0.05.

** P ≤ 0.01.

**Table 2.** Effect of type of file and chemical materials in time.

Chemical materials	Mean ± SE of time (sec.)		LSD	P-value
	Edge file	Fanta file		
Glycerin	8.85 ± 0.32 A a	4.48 ± 0.14 A b	0.725 **	0.0001
EDTA	5.61 ± 0.29 B a	3.70 ± 0.22 B b	0.764 **	0.0001
Sodium hypo-chloride	5.45 ± 0.09 B a	3.63 ± 0.24 B b	0.543 **	0.0001
LSD	0.741 **	0.598 **	---	
P-value	**0.0001**	**0.010**		

Means with different big letters in the same column and small letters in the same row are significantly different.

** P ≤ 0.01.

**Figure 2.**
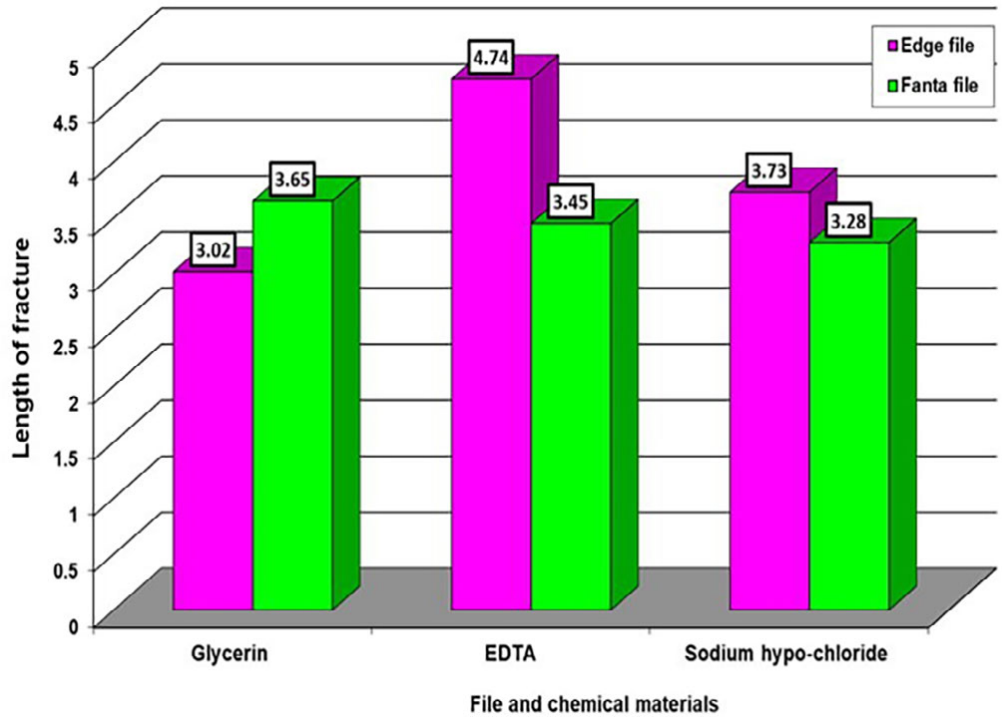
The effect of types of file and chemical material on length of fractures.

**Figure 3.**
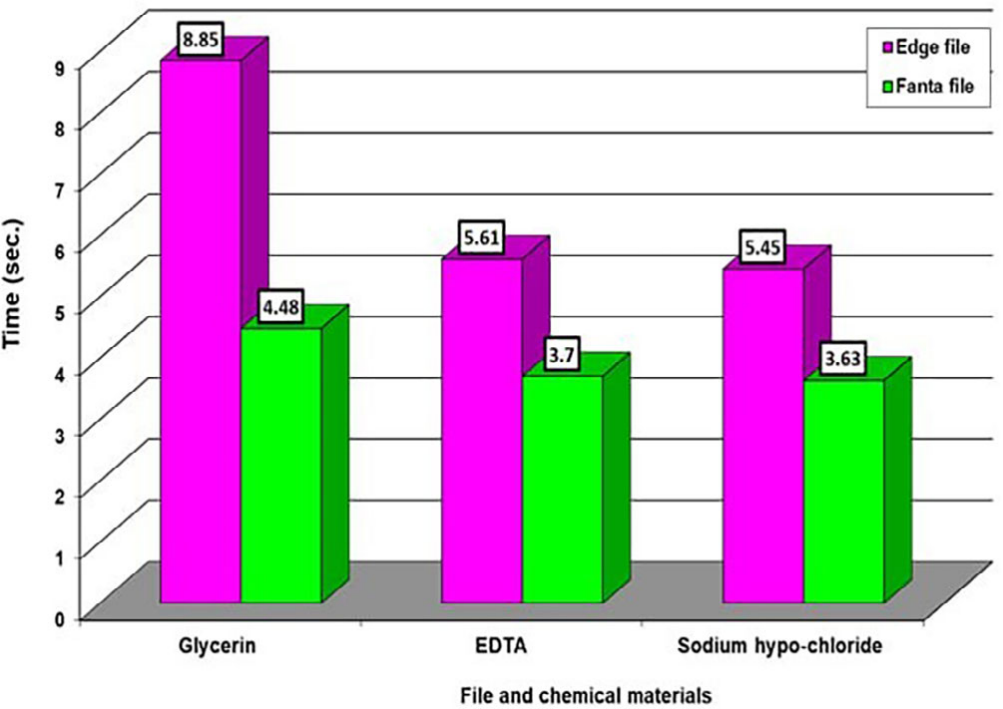
The effect of types of file and chemical material on time.

In Table 1 we observed notable variations in the impact of three materials on the edge file. Specifically, there were high significant differences noted for the material EDTA, followed by NaOH and then Glycerin, respectively. However, there were no significant differences observed among the chemical materials on the Fanta file. In Table 2, we observed very significant differences between the effect of Glycerin and the other two types of chemical materials, while there were no significant differences between the two chemical materials when compared with each other on the two types of files. Averages that carry different letters in Tables 1 and 2 are significantly different, and averages that carry similar letters do not differ significantly. The highest average takes the letter A and so on downwards. If you find an average that takes two letters like ab, this is no different neither from the average that carries a nor from the average that carries b. as appeared in Figures 2 and 3.

## Discussion

Despite considerable advancements in NiTi instrument design and technology, the persistence of NiTi instrument failure during root canal therapy remains a significant issue. These instruments are prone to fracture without apparent symptoms of permanent distortion. Even with the development of new endodontic instrument generations employing various manufacturing processes, the possibility of instrument fracture still exists.

Every file system must undergo disinfection for infection control before reuse. While performing their functions, instruments are exposed to commonly used irrigants such as NaOCl, which fills the canals during instrumentation. Although various research efforts have assessed the cyclic fatigue resistance of different file systems, the effects of autoclaving and exposure during operation on various file systems’ properties may vary. Consequently, this study contrasts multiple file systems, utilizing diverse manufacturing technologies, for cyclic fatigue resistance after exposure to NaOCl, glycine, and EDTA.

The study aims to evaluate how irrigation affects the resistance to cyclic fatigue of two rotary endodontic instruments. Chemically active irrigation solutions are used during the clinical shaping of curved root canals. Surface interactions between the file and canal walls during this process could lead to corrosion or surface roughness, potentially resulting in fissures and cyclic fatigue of the file ( Hasegawa *et al.*, 2014).

Various researchers have employed different methods, including pre-immersion of NiTi files in an irrigation solution or testing files for cyclic fatigue in synthetic oil, to evaluate the fatigue failure process ( Pedullà *et al.*, 2014).

The interaction between the file and the irrigation solutions while the file is rotating in the irrigation solution is not taken into account by this design. In additional studies, the files were evaluated in an irrigation solution bath ( Elnaghy and Elsaka, 2017). However, the relationship between the file and the canal walls was not taken into account. The files were tested while rotating in a curved glass tube filled with the irrigation fluid as part of the current study’s specially created apparatus, which duplicates the clinical settings. To prevent galvanic corrosion from happening during testing in metal to or in contact with metal pins, as was stated in the previous research, a heat-resistant curved glass tube was employed ( Shen *et al.*, 2012). Glass tubes, which are not found in natural teeth, may have several drawbacks that come from chemical reactions with various irrigation solutions and should not be extrapolated clinically.

Theoretically, employing aqueous media will allow the heat generated by friction to evaporate and gradually lengthen the fatigue lifetime of NiTi files. However, the current study’s findings, which are in agreement with some reports ( Shen *et al.*, 2012; Hasegawa *et al.*, 2014; Pedullà *et al.*, 2011) and disagreement with others, show that the NCF of instruments tested in dry conditions without lubricant or coolant was significantly different from those tested in EDTA, Glycine, and NaOCl groups ( Berutti *et al.*, 2006; Elnaghy and Elsaka, 2017; Peters *et al.*, 2007). These contradictory results could be the consequence of one of two factors.

The first is that these aqueous solutions may hurtan an adverse effect on cyclic fatigue due to their propensity to produce corrosion and the roughness of the NiTi alloy surface. As a result, the fatigue life did not improve. The second factor is that fatigue testing occurred in a location with minimal cyclic fatigue. Regarding the first factor, prior studies indicated that immersion in NaOCl and EDTA could result in file corrosion ( Berutti *et al.*, 2006; Peters *et al.*, 2007).

However, in these studies, the file and shank were fully submerged for a long period of time, which led to galvanic corrosion, which is clinically inapplicable. Recent studies, which concur with the present findings, have shown that immersion does not, on the other hand, promote corrosion ( Shen *et al.*, 2012; Pedullà *et al.*, 2011) or exhibit any adverse impact on cyclic fatigue even with an increased surface roughness of the files.

The second theory appears more believable. The NCF in the existing configuration was in the low-cyclic fatigue range ( Cheung *et al.*, 2007; Tobushi *et al.*, 1997). This amount of time is insufficient to demonstrate how the various circumstances interact. When the number of cycles fell within the low-cyclic fatigue life, Shen *et al.* (2012) showed no difference in NCF between the NCF of various files in dry conditions and a bath of irrigation solutions including Glycine, EDTA, and NaOCl. In contrast to dry conditions, liquid media has shown improvement in cycle fatigue in long fatigue life. In the landmark study by Tobushi *et al.* (1997), the researchers discovered that in areas of mild tiredness, there was no difference between wet and dry environments.

Similarly, to this, Pedullà *et al.* (2014) demonstrated that submerging NiTi files in EDTA and NaOCl had no detrimental effects on the NCF. On the other hand, testing WaveOne Gold in the air revealed better fatigue resistanceto fatigue compared to aqueous media, according to Elnaghy and Elsaka (2017). The methodology, instrument design, and type of motion employed may all be to fault for the inconsistent outcomes.

The degree of curvature is another element that could obscure the effect of irrigation solutions on cycle fatigue. It is understood that a file moving through a steep curve experiences a significant strain amplitude, hastening the failure process ( Cheung *et al.*, 2007). A 60° curvature was used in the existing configuration. As a result, there wasn’t enough time to demonstrate how a different environment (a low-fatigue region) affected things.

Hasegawa *et al.* (2014) revealed that when evaluated in a 60° curved environment, three separate files examined in various conditions had similar NCF results. When the files were examined under a 30° arc of curvature, water increased the NFC of the files, but a different NFC was recorded. So, it stands to reason that aqueous solution performance would be greater with longer testing periods.

It’s interesting to note that two fracture sites in the NaOCl group spread toward the file’s center after being launched from opposing cutting edges. This might be connected to surface roughness, which could serve as a site for crack propagation. Thus, many cracks might account for the group’s lower cycle count. The short testing period is the study’s main drawback. The impact of irrigation solutions in areas with high cyclic fatigue should be evaluated. Additionally, analyzing the frictional forces that each solution generates and how they might affect the NCF may be helpful. The fracture’s length was determined to understand how the overload zones manifested dimpled rupture evidence. This rupture occurred due to the coalescence of microvoids in the overload zone, ultimately leading to the ductile fracture of the instruments.

## Conclusion

The comparison between the two instruments that were tested revealed that NaOCl, glycine, and EDTA as chemical materials appeared to have noticeably different cycle fatigue resistance for various lengths of fractures and timeframes. The cyclic fatigue of NiTi files may be impacted by the irrigation environment. Chemical materials enhanced file NCF. However, due to the brief testing period (low-cyclic fatigue region), all other settings did not demonstrate any differences. Since cyclic fatigue takes far longer to develop than it does to shape actual teeth, the results of this *in vitro* study should not be directly applied to clinical situations. So, it’s important to proceed carefully when drawing clinical conclusions. However, further research is required to fully understand the various variables that can impact an instrument’s cyclic fatigue resistance, fracture modes, and novel apparatus designs that share more properties with root dentine. Further research is therefore required to support the results of the current study.

## Contributor role

Conceptualization: Mohammed Hamoudi Alsunboli

Data Curation: Sally Saad Ali Ihsan

Formal Analysis: Sally Saad Ali Ihsan

Funding Acquisition: Mohammed Hamoudi Alsunboli

Investigation: Duha Qais Sabah

Methodology: Duha Qais Sabah

Project Administration: Sally Saad Ali Ihsan

Resources: Mohammed Hamoudi Alsunboli

Software: Duha Qais Sabah

Supervision: Duha Qais Sabah

Validation: Mohammed Hamoudi Alsunboli

Visualization: all authors

Writing – Original Draft Preparation: all authors

Writing – Review & Editing: all authors

## Data Availability

Zenodo. Basic data that show effect of irrigation on the cyclic fatigue resistance of the two rotary endodontic instruments. DOI: https://doi.org/10.5281/zenodo.7600416 ( Alsunboli, 2023). This project contains the following underlying data:
• Edge file with glycerin • Edge file with EDTA • Edge file with sodium hypochlorite • Fanta file WITH glycerin • Fanta file with EDTA • Fanta file with sodium hypochlorite Data are available under the terms of the Creative Commons Zero “No rights reserved” data waiver (CC0 1.0 Public domain dedication).

## References

[ref1] Alsunboli MH : Basic data that show effect of irrigation on the cyclic fatigue resistance of the two rotary endodontic instruments. *Zenodo.* 2023. 10.5281/zenodo.7600416

[ref2] Arens FC Hoen MM Steiman HR : Evaluation of single-use rotary nickel-titanium instruments. *J. Endod.* 2003;29:664–666. 10.1097/00004770-200310000-00013 14606792

[ref3] Berutti E Angelini E Rigolone M : Influence of sodium hypochlorite on fracture properties and corrosion of proTaper rotary instruments. *Int. Endod. J.* 2006;39:693–699. 10.1111/j.1365-2591.2006.01134.x 16916358

[ref4] Berutti E Chiandussi G Paolino DS : Canal shaping with WaveOne reciprocating files and Pro Taper system: a comparative study. *J. Endod.* 2012a;38:505–509. 10.1016/j.joen.2011.12.040 22414838

[ref5] Berutti E Paolino DS Chiandussi G : Root canal anatomy preservation of WaveOne reciprocating files with or without glyde path. *J. Endod.* 2012b;38:101–104. 10.1016/j.joen.2011.09.030 22152630

[ref6] Bhagabati N Yadav S Talwar S : An *in vitro* cyclic fatigue analysis of different endodontic nickel-titanium rotary instruments. *J. Endod.* 2012;38:515–518. 10.1016/j.joen.2011.12.034 22414840

[ref7] Chen JL Messer HH : A comparison of stainless steel hand and rotary nickel- titanium instrumentation using a silicone impression technique. *Aust. Dent. J.* 2002;47:12–20. 10.1111/j.1834-7819.2002.tb00297.x 12035951

[ref8] Cheung G Ko DH Chung S : Fatigue testing of a NiTi rotary instrument. Part 2: Fractographic analysis. *Int. Endod. J.* 2007;40:619–625. 10.1111/j.1365-2591.2007.01256.x 17511786

[ref9] Elnaghy AM Elsaka SE : Effect of sodium hypochlorite and saline on cyclic fatigue resistance of WaveOne Gold and Reciproc reciprocating instruments. *Int. Endod. J.* 2017;50:991–998. 10.1111/iej.12712 27770436

[ref10] Estrela CR Estrela C Reis C : Control of microorganisms *in vitro* by endodontic irrigants. *Braz. Dent. J.* 2003;14(3):187–192. 10.1590/S0103-64402003000300009 15057395

[ref11] Gambarini G Grande NM Plotino G : Fatigue resistance of engine-driven rotary nickel–titanium instruments produced by new manufacturing methods. *J. Endod.* 2008;34:1003–1005. 10.1016/j.joen.2008.05.007 18634935

[ref12] Grande NM Plotino G Pecci R : Cyclic fatigue resistance and three-dimensional analysis of instruments from two nickel-titanium rotary systems. *Int. Endod. J.* 2006;39(10):755–763. 10.1111/j.1365-2591.2006.01143.x 16948660

[ref13] Hasegawa Y Goto S Ogura H : Effect of EDTA solution on corrosion fatigue of Ni-Ti files with different shapes. *Dent. Mater. J.* 2014;33:415–421. 10.4012/dmj.2013-283 24786349

[ref14] Hulsmann M Peters OA Dummer PMH : Mechanical preparation of root canals: shaping goals, techniques and means. *Endod. Top.* 2005;10:30–76. 10.1111/j.1601-1546.2005.00152.x

[ref15] Pedullà E Franciosi G Ounsi HF : Cyclic fatigue resistance of nickel-titanium instruments after immersion in irrigant solutions with or without surfactants. *J. Endod.* 2014;40:1245–1249. 10.1016/j.joen.2014.02.005 25069942

[ref16] Pedullà E Grande NM Plotino G : Cyclic fatigue resistance of three different nickel-titanium instruments after immersion in sodium hypochlorite. *J. Endod.* 2011;37:1139–1142. 10.1016/j.joen.2011.04.008 21763909

[ref17] Peters OA Roehlike JO Baumann MA : Effect of immersion in sodium hypochlorite on torque and fatigue resistance of nickel-titanium instruments. *J. Endod.* 2007;33:589–593. 10.1016/j.joen.2007.01.007 17437879

[ref18] Plotino G Grande NM Sorci E : A comparison of cyclic fatigue between used and new Mtwo Ni-Ti rotary instruments. *Int. Endod. J.* 2006;39:716–723. 10.1111/j.1365-2591.2006.01142.x 16916361

[ref19] Plotino G Grande NM Cordaro M : A review of cyclic fatigue testing of nickel-titanium rotary instruments. *J. Endod.* 2009;35:1469–1476. 10.1016/j.joen.2009.06.015 19840633

[ref20] Plotino G Grande NM Melo MC : Cyclic fatigue of NiTi rotary instruments in a simulated apical abrupt curvature. *Int. Enndodont. J.* 2010;43:226–230. 10.1111/j.1365-2591.2009.01668.x 20158534

[ref21] SAS : *Statistical Analysis System, User’s Guide. Statistical. Version 9.* 1st ed. Cary NC USA: SAS. Inst. Inc.;2018.

[ref22] Schafer E Schulz-Bongert U : Tulus G: Comparison of hand stainless steel and nickel titanium rotary instrumentation: a clinical study. *J. Endod.* 2004;30:432–435. 10.1097/00004770-200406000-00014 15167474

[ref23] Schneider SW : A comparison of canal preparations in straight and curved root canals. *Oral Surg. Oral Med. Oral Pathol.* 1971;32(2):271–275. 10.1016/0030-4220(71)90230-1 5284110

[ref24] Shen Y Qian W Abtin H : Effect of environment on fatigue failure of controlled memory wire nickel-titanium rotary instruments. *J. Endod.* 2012;38:376–380. 10.1016/j.joen.2011.12.002 22341078

[ref25] Sonntag D Heithecker K : *Korrosion von Nickel-Titan-Instrumenten. Endodontie.* 2006;15:23–30.

[ref26] Tobushi H Hachisuka T Yamada S : Rotating-bending fatigue of a TiNi shape-memory alloy wire. *Mech. Mater.* 1997;26:35–42. 10.1016/S0167-6636(97)00019-7

[ref27] Torabinejad M Walton RE :2009; *Endodontics: principles and practice.* 4th ed. Missouri: St. Louis.

[ref28] Varela-Patino P Ibanez-Parraga A Rivas-Mundina B : Alternating versus continuous rotation: a comparative study of the effect on instrument life. *J. Endod.* 2010;36:157–159. 10.1016/j.joen.2009.09.023 20003957

[ref29] Wong J Cheung GSP Lee AHC : PROMs following Root Canal Treatment and Surgical Endodontic Treatment. *Int. Dent. J.* 2023;73:28–41. 10.1016/j.identj.2022.06.015 35871899 PMC9875275

[ref30] Yılmaz K Uslu G Özyürek T : *In vitro* comparison of the cyclic fatigue resistance of HyFlex EDM, One G, and ProGlider nickel titanium glide path instruments in single and double curvature canals. *Restorative Dent. Endodont.* 2017;42:282–289. 10.5395/rde.2017.42.4.282 29142876 PMC5682144

