# Supplementary material for: The effect of canal curvature on cyclic fatigue resistance of rotary instruments using different irrigation materials ( in vitro study)
Source: F1000Res. 2023 Apr 27;12:449. [Version 1] doi: 10.12688/f1000research.130249.1 (PMC11078266; doi:10.12688/f1000research.130249.1)
Supplement: Peer review history

# Response

**Published:** 2024-01-28

**Authors:** Susan F. K. Alsudani

**Affiliations:** Al-Bayan University , College of Dentistry, Iraq

thank you Dr. Rashid ElAbed for these valuable comments

 I make grammar revisions for all articles and add your notes with highlighting

 thank you

**The effect of canal curvature on cyclic fatigue resistance of rotary instruments using different irrigation materials ( in vitro study)**

 Mohammed Hamoudi Alsunboli ^1*^ , Sally Saad Ali Ihsan ^2^* , Duha Qais Sabah ^3^*

 *Al-Bayan University , College of Dentistry

 1- m.hamoudi@albayan.edu.iq, 2- sally.saad@albayan.edu.iq,3- dhuha.q@albayan.edu.iq.

**Abstract**

**Background: **The mechanical qualities of Ni Ti are crucial because they give the files their flexibility and enable us to prepare curved and double-curved canals with more ease. It happens frequently for instruments to separate during canal preparation, and cyclic fatigue (metal fatigue) is a frequent cause.

**Aim of study:** This study aimed to assess how irrigation affected the two rotary endodontic instruments' cyclic fatigue resistance.

**Methods**: The Edge File and Fanta File rotary endodontic instrument groups were chosen. Each group (n = 42) was split into 3 subgroups (n = 14 each), one receiving NaOH, one Glycine, and one EDTA treatment. The number of cycles to failure (NCF) was determined after each subgroup underwent testing for cyclic fatigue resistance.

**Results**: The result appeared different significant between the two group and sub group with the different materials that used with it with the length of fractures and time that recorded in each group.

**Conclusion: **NaOCl, glycine, and EDTA as chemical materials appeared to have considerably varied cycle fatigue resistance for various lengths of fractures and durations, according to the comparison between the two evaluated instruments **.**

**Keywords: **cyclic fatigue, rotation, fracture, M wire, autoclave, irrigation, curve canal

**Introduction**

The success of endodontic therapy is associated with the cleansing and shape of the root system, which is impacted by mechanical elements such the preparation system and preparation process. The effectiveness of chemical agents with irrigation solutions in tissue solution, germination, and residue removal is covered next. (Estrela et al.,2003).

The durability of a rotary endodontic tool inside an angulated root canal is determined by the extent of flexing it experiences, influencing its longevity before succumbing to fatigue. A torsional fracture arises when the instrument shaft still rotates with the endodontic file's tip, or a fragment of it, lodged within a canal. In such cases, the metal surpasses its elastic limits, resulting in plastic deformation and eventual fracture (Berutti et al., 2012).

In modern endodontics, NiTi rotary devices universally shape root canals (Torabinejad and Walton, 2009). They exhibit superior flexibility and cutting efficiency compared to standard stainless steel files (Schafer et al., 2004). The remarkable flexibility simplifies achieving the desired tapering root canal form with a reduced risk of canal transportation (Chen and Messer, 2002). Despite these advantages, NiTi instruments are susceptible to separation (Arens et al., 2003), primarily due to wear and torsional shear forces (Varela-Patino et al., 2010; Berutti et al., 2012). Cyclic fatigue occurs during rotating instrumentation in a curved canal, subjecting the instrument to repeated cycles of compression and tension (Chen and Messer, 2002).

Operational speed, metal surface treatment, and metallurgical characterization of NiTi alloys are among the variables studied for potential influences on the fatigue resistance of NiTi rotary files (Gambarini et al., 2008).

Canal curvature, attributed to flexural loads and cyclic fatigue, is considered the primary cause of instrument failure (Hulsmann et al., 2005). Corrosion in the presence of an irrigating solution is another factor that may diminish resistance to fatigue fracture (Sonntag and Heithecker, 2006). The gold standard for tissue disintegration and disinfection involves irrigating root canals with NaOCl and EDTA (Torabinejad and Walton, 2009). NiTi instruments, when used in root canal instrumentation, come into contact with these irrigating solutions.

Consequently, corrosion patterns, involving the selective removal of nickel from the surface, may lead to micro pitting, compromising the structural integrity of the instrument. Clinicians have limited options to mitigate such stresses (Berutti et al., 2012). This study aims to evaluate the impact of irrigation on the cyclic fatigue resistance of two rotary endodontic devices.

**Material and Methods**

For this study, the Edge File (0.25/0.6) and Fanta file (0.25/0.6) groups of rotary endodontic instruments were selected. Each group comprised three subgroups, resulting in a total of 42 samples (n = 42), with 14 samples in each subgroup.

The subgroups were designated as follows:

Subgroup 1: Treated with glycerin

Subgroup 2: Treated with EDTA

Subgroup 3: Treated with sodium hypochlorite

Considering the diverse canal shapes found in natural teeth, conducting cyclic fatigue tests consistently with natural teeth is impractical.

This study employed artificial canals constructed from fixed tapered stainless steel. These canals were placed in a water bath at 37ºC, specifically designed for this research. The synthetic canals featured regular (60º) curvature angles, a 5mm radius of curvature, and a gradual reduction in the width of the coronal portion from 1.5mm to 1mm at its end (Plotino and others, 2009).

The testing followed the manufacturer-recommended settings for the rotary system (500 Rpm, 2.5 N torque) for both Fanta and Edge systems. The file tip, set to its full working length, was positioned 5-7mm from the center of the simulated curvature (19mm). The working area spanned 25mm, and all files were new. The cyclic fatigue tests involved rotating the file as static rest freely inside the tapered section of the artificial canal, simulating the file's restraint in the canal's curved section (Schneider, 1971).

To facilitate hand-piece movement and file insertion, the dental hand-piece was mounted on a wooden block, ensuring uniform file depth placement and three-dimensional alignment. A transparent plastic sheet prevented file slippage and allowed researchers to observe file usage and fracture development. The wooden block was secured to the stainless steel block to minimize movement (Yılmaz et al., 2017).

Glycerin was used to reduce heat and friction, packed inside the artificial canal before each file's insertion. The files were activated inside the canals using the cordless endodontic hand-piece (ENDOMAX PLUS), with concurrent video recording to enhance productivity and reduce errors (Bhagabati et al., 2012). The number of cycles to failure (NCF) was described by the equation: "NCF = Speed (RPM) X Time (T) to fracture in minutes." The study's armamentarium is depicted in Fig(1)

**Fig (1): The armamentarium used in this study**

After being removed from the solutions, each file was rinsed with bi-distilled water to counteract the effects of NaOCl, dried, given an ID number, and stored in glass vials.

Then, using a mechanical device created expressly for the job and capable of simulating the conditions of an instrument encased in a curved canal, instruments from all three groups and each brand were put through cyclic fatigue testing. (Grande et al. 2006, Plotino et al. 2009).

The apparatus was connected to the same dynamic immersion programs and motor set. This made it possible for the endodontic instruments to freely reciprocate and maintain constant pressure inside a stainless steel artificial channel. To make the artificial canal, the instrument's dimensions and taper were reproduced. When the file made contact with the canal walls in the simulated canal, a special high-flow synthetic lubricant (Super Oil; Singer Co Ltd, Elizabethport, NJ, USA) was sprayed into the canal to lessen friction. The time to fracture (TtF) for each instrument, measured in seconds from the start of the test until the point of breakage, was recorded and registered using a chronometer to the nearest whole number. The TtF was the dependent variable, whereas the type of files used and the immersion conditions were independent factors.

**Statistical Analysis:**

The Statistical Analysis System- SAS (2018) application was used to determine the effects of various factors on the research parameters. The least significant difference (LSD) test was used in this investigation to compare the means in a significant manner (ANOVA).

**Results**

Descriptive statistics of TtF for the each file are summarized in Table (1 and 2).

**Table 1: Effect of  type of File and chemical materials in Length of**

**Chemical materials**

**Mean ± SE of Length of fracture**

**LSD**

**P-value**

**Edge file**

**Fanta file**

**Glycerin**

**3.02 ±0.17**

**C     b**

**3.65 ±0.16**

**A     a**

0.490 *

**0.0136**

**EDTA**

**4.74 ±0.11**

**A     a**

**3.45 ±0.09**

**A     b**

0.304 **

**0.0001**

**Sodium hypo-chloride**

**3.73 ±0.12**

**B     a**

**3.28 ±0.16**

**A     b**

0.421 *

**0.0371**

**LSD**

0.397 **

**0.413 NS**

**---**

**P-value**

**0.0001**

**0.199**

Means with different big letters in the same column and small letters in the same row are significantly different ,  * (P≤0.05),  ** (P≤0.01).

**Table 2: Effect of  type of File and chemical materials in Time**

**Chemical materials**

**Mean ± SE of Time (sec.)**

**LSD**

**P-value**

**Edge file**

**Fanta file**

**Glycerin**

**8.85 ±0.32**

**A     a**

**4.48 ±0.14**

**A     b**

0.725 **

**0.0001**

**EDTA**

**5.61 ±0.29**

**B     a**

**3.70 ±0.22**

**B     b**

0.764 **

**0.0001**

**Sodium hypo-chloride**

**5.45 ±0.09**

**B     a**

**3.63 ±0.24**

**B     b**

0.543 **

**0.0001**

**LSD**

0.741 **

0.598 **

**---**

**P-value**

**0.0001**

**0.010**

Means with different big letters in the same column and small letters in the same row are significantly different ,  ** (P≤0.01).

In the table (1), we notice different significant differences in the effect of three materials on the edge file, which appeared a high significant differences for the material EDTA, then NaOH, then Glycerin in respectively, while there are no significant differences of the chemical materials on the Fanta file. While In the table (2), we notice very high significant differences between the effect of glycerin and the other two types of chemical materials, while appeared no significant differences between the two chemical materials when compared with each other on the two types of files.

Note:- Averages that carry different letters in table (1 and 2) are significantly different, and averages that carry similar letters do not differ significantly. The highest average takes the letter a and so on downwards. If you find an average that takes two letters like ab, this is no different neither from the average that carries a nor from the average that carries b. as appeared in figure (2 and 3)

**Figure (2) the effect of types of file and chemical material on length of fractures**

**Figure (3) the effect of types of file and chemical material on time**

**Discussion**

Despite considerable advancements in NiTi instrument design and technology, the persistence of NiTi instrument failure during root canal therapy remains a significant issue. These instruments are prone to fracture without apparent symptoms of permanent distortion. Even with the development of new endodontic instrument generations employing various manufacturing processes, the possibility of instrument fracture still exists.

Every file system must undergo disinfection for infection control before reuse. While performing their functions, instruments are exposed to commonly used irrigants such as NaOCl, which fills the canals during instrumentation. Although various research efforts have assessed the cyclic fatigue resistance of different file systems, the effects of autoclaving and exposure during operation on various file systems' properties may vary. Consequently, this study contrasts multiple file systems, utilizing diverse manufacturing technologies, for cyclic fatigue resistance after exposure to NaOCl, glycine, and EDTA.

The study aims to evaluate how irrigation affects the resistance to cyclic fatigue of two rotary endodontic instruments. Chemically active irrigation solutions are used during the clinical shaping of curved root canals. Surface interactions between the file and canal walls during this process could lead to corrosion or surface roughness, potentially resulting in fissures and cyclic fatigue of the file (Hasegawa et al., 2014).

Various researchers have employed different methods, including pre-immersion of NiTi files in an irrigation solution or testing files for cyclic fatigue in synthetic oil, to evaluate the fatigue failure process (Pedulla et al., 2014).

The interaction between the file and the irrigation solutions while the file is rotating in the irrigation solution is not taken into account by this design. In additional studies, the files were evaluated in an irrigation solution bath (Elnaghy and Elsaka, 2017). However, the relationship between the file and the canal walls was not taken into account.

The files were tested while rotating in a curved glass tube filled with the irrigation fluid as part of the current study's specially created apparatus, which duplicates the clinical settings. To prevent galvanic corrosion from happening during testing in metal to or in contact with metal pins, as was stated in the previous research, a heat-resistant curved glass tube was employed (Shen et al., 2012). Glass tubes, which are not found in natural teeth, may have several drawbacks that come from chemical reactions with various irrigation solutions and should not be extrapolated clinically.

Theoretically, employing aqueous media will allow the heat generated by friction to evaporate and gradually lengthen the fatigue lifetime of NiTi files. However, the current study's findings, which are in agreement with some reports (Shen et al., 2012; Hasegawa et al., 2014; Pedulla et al., 2011) and disagreement with others, show that the NCF of instruments tested in dry conditions without lubricant or coolant was significantly different from those tested in EDTA, Glycine, and NaOCl groups (Berutti et al., 2006; Elnaghy and Elsaka, 2017; Peters et al., 2007) These contradictory results could be the consequence of one of two factors.

The first is that these aqueous solutions may hurtan an adverse effect on cyclic fatigue due to their propensity to produce corrosion and the roughness of the NiTi alloy surface. As a result, the fatigue life did not improve. The second factor is that fatigue testing occurred in a location with minimal cyclic fatigue. Regarding the first factor, prior studies indicated that immersion in NaOCl and EDTA could result in file corrosion (Berutti et al., 2006; Peter et al., 2007).

However, in these studies, the file and shank were fully submerged for a long period of time, which led to galvanic corrosion, which is clinically inapplicable. Recent studies, which concur with the present findings, have shown that immersion does not, on the other hand, promote corrosion (Shen et al., 2012; Pedulla et al., 2011) or exhibit any adverse impact on cyclic fatigue even with an increased surface roughness of the files.

The second theory appears more believable. The NCF in the existing configuration was in the low-cyclic fatigue range (Cheung et al., 2007; Tobushi et al., 1997). This amount of time is insufficient to demonstrate how the various circumstances interact. When the number of cycles fell within the low-cyclic fatigue life, Shen et al. (2012) showed no difference in NCF between the NCF of various files in dry conditions and a bath of irrigation solutions including Glycine, EDTA, and NaOCl. In contrast to dry conditions, liquid media has shown improvement in cycle fatigue in long fatigue life. In the landmark study by Tobushi et al. (1997), the researchers discovered that in areas of mild tiredness, there was no difference between wet and dry environments.

Similarly to this, Pedullà et al. (2014) demonstrated that submerging NiTi files in EDTA and NaOCl had no detrimental effects on the NCF. On the other hand, testing WaveOne Gold in the air revealed better fatigue resistanceto fatigue compared to aqueous media, according to Elnaghy and Elsaka (2017). The methodology, instrument design, and type of motion employed may all be to fault for the inconsistent outcomes.

The degree of curvature is another element that could obscure the effect of irrigation solutions on cycle fatigue. It is understood that a file moving through a steep curve experiences a significant strain amplitude, hastening the failure process (Cheung et al., 2007). A 60° curvature was used in the existing configuration. As a result, there wasn't enough time to demonstrate how a different environment (a low-fatigue region) affected things.

Hasegawa et al. (2014) revealed that when evaluated in a 60° curved environment, three separate files examined in various conditions had similar NCF results. When the files were examined under a 30° arc of curvature, water increased the NFC of the files, but a different NFC was recorded. So it stands to reason that aqueous solution performance would be greater with longer testing periods.

It's interesting to note that two fracture sites in the NaOCl group spread toward the file's center after being launched from opposing cutting edges. This might be connected to surface roughness, which could serve as a site for crack propagation. Thus, many cracks might account for the group's lower cycle count. The short testing period is the study's main drawback. The impact of irrigation solutions in areas with high cyclic fatigue should be evaluated. Additionally, analyzing the frictional forces that each solution generates and how they might affect the NCF may be helpful.

**Conclusion**

The comparison between the two instruments that were tested revealed that NaOCl, glycine, and EDTA, as chemical materials, exhibited noticeably different cyclic fatigue resistance for various lengths of fractures and timeframes. The cyclic fatigue of NiTi files may be influenced by the irrigation environment. Chemical materials enhanced the files' Number of Cycles to Failure (NCF). However, due to the brief testing period (in the low-cyclic fatigue region), all other settings did not show any differences. Since cyclic fatigue takes much longer to develop than shaping actual teeth, the results of this in vitro study should not be directly applied to clinical situations. Therefore, caution is essential when drawing clinical conclusions. Nevertheless, further research is necessary to fully understand the various variables that can impact an instrument's cyclic fatigue resistance, fracture modes, and novel apparatus designs that share more properties with root dentine. Additional research is, therefore, required to validate and support the results of the current study.

# Response

**Published:** 2024-02-18

**Authors:** Susan F. K. Alsudani

**Affiliations:** Al-Bayan University , College of Dentistry, Iraq

thank you Dr. Shehabeldin Saber for these valuable comments

 its static test and I do it in a bath of 37 Cº

 I make grammar revisions for all articles and add your notes with highlighting

 thank you

**Abstract**

**Background: **The mechanical qualities of Ni Ti are crucial because they give the files their flexibility and enable us to prepare curved and double-curved canals with more ease. It happens frequently for instruments to separate during canal preparation, and cyclic fatigue (metal fatigue) is a frequent cause.

**Aim of study:** This study aimed to assess how irrigation affected the two rotary endodontic instruments' cyclic fatigue resistance.

**Methods**: The Edge File and Fanta File rotary endodontic instrument groups were chosen. Each group (n = 42) was split into 3 subgroups (n = 14 each), one receiving NaOH, one Glycine, and one EDTA treatment. The number of cycles to failure (NCF) was determined after each subgroup underwent testing for cyclic fatigue resistance.

**Results**: The result appeared different significant between the two group and sub group with the different materials that used with it with the length of fractures and time that recorded in each group.

**Conclusion: **NaOCl, glycine, and EDTA as chemical materials appeared to have considerably varied cycle fatigue resistance for various lengths of fractures and durations, according to the comparison between the two evaluated instruments **.**

**Keywords: **cyclic fatigue, rotation, fracture, M wire, autoclave, irrigation, curve canal

**Introduction**

 The success of endodontic therapy is associated with the cleansing and shape of the root system, which is impacted by mechanical elements such the preparation system and preparation process. The effectiveness of chemical agents with irrigation solutions in tissue solution, germination, and residue removal is covered next. (Estrela et al.,2003).

 The durability of a rotary endodontic tool inside an angulated root canal is determined by the extent of flexing it experiences, influencing its longevity before succumbing to fatigue. A torsional fracture arises when the instrument shaft still rotates with the endodontic file's tip, or a fragment of it, lodged within a canal. In such cases, the metal surpasses its elastic limits, resulting in plastic deformation and eventual fracture (Berutti et al., 2012).

 In modern endodontics, NiTi rotary devices universally shape root canals (Torabinejad and Walton, 2009). They exhibit superior flexibility and cutting efficiency compared to standard stainless steel files (Schafer et al., 2004). The remarkable flexibility simplifies achieving the desired tapering root canal form with a reduced risk of canal transportation (Chen and Messer, 2002). Despite these advantages, NiTi instruments are susceptible to separation (Arens et al., 2003), primarily due to wear and torsional shear forces (Varela-Patino et al., 2010; Berutti et al., 2012). Cyclic fatigue occurs during rotating instrumentation in a curved canal, subjecting the instrument to repeated cycles of compression and tension (Chen and Messer, 2002).

 Operational speed, metal surface treatment, and metallurgical characterization of NiTi alloys are among the variables studied for potential influences on the fatigue resistance of NiTi rotary files (Gambarini et al., 2008).

 Canal curvature, attributed to flexural loads and cyclic fatigue, is considered the primary cause of instrument failure (Hulsmann et al., 2005). Corrosion in the presence of an irrigating solution is another factor that may diminish resistance to fatigue fracture (Sonntag and Heithecker, 2006). The gold standard for tissue disintegration and disinfection involves irrigating root canals with NaOCl and EDTA (Torabinejad and Walton, 2009). NiTi instruments, when used in root canal instrumentation, come into contact with these irrigating solutions.

 Consequently, corrosion patterns, involving the selective removal of nickel from the surface, may lead to micro pitting, compromising the structural integrity of the instrument. Clinicians have limited options to mitigate such stresses (Berutti et al., 2012). This study aims to evaluate the impact of irrigation on the cyclic fatigue resistance of two rotary endodontic devices.

**Material and Methods**

 For this study, the Edge File (0.25/0.6) and Fanta file (0.25/0.6) groups of rotary endodontic instruments were selected. Each group comprised three subgroups, resulting in a total of 42 samples (n = 42), with 14 samples in each subgroup.

 The subgroups were designated as follows:

 Subgroup 1: Treated with glycerin

 Subgroup 2: Treated with EDTA

 Subgroup 3: Treated with sodium hypochlorite

 Considering the diverse canal shapes found in natural teeth, conducting cyclic fatigue tests consistently with natural teeth is impractical.

 This study employed artificial canals constructed from fixed tapered stainless steel. These canals were placed in a water bath at 37ºC, specifically designed for this research. The synthetic canals featured regular (60º) curvature angles, a 5mm radius of curvature, and a gradual reduction in the width of the coronal portion from 1.5mm to 1mm at its end (Plotino and others, 2009).

 The testing followed the manufacturer-recommended settings for the rotary system (500 Rpm, 2.5 N torque) for both Fanta and Edge systems. The file tip, set to its full working length, was positioned 5-7mm from the center of the simulated curvature (19mm). The working area spanned 25mm, and all files were new. The cyclic fatigue tests involved rotating the file as static rest freely inside the tapered section of the artificial canal, simulating the file's restraint in the canal's curved section (Schneider, 1971).

 To facilitate hand-piece movement and file insertion, the dental hand-piece was mounted on a wooden block, ensuring uniform file depth placement and three-dimensional alignment. A transparent plastic sheet prevented file slippage and allowed researchers to observe file usage and fracture development. The wooden block was secured to the stainless steel block to minimize movement (Yılmaz et al., 2017).

 Glycerin was used to reduce heat and friction, packed inside the artificial canal before each file's insertion. The files were activated inside the canals using the cordless endodontic hand-piece (ENDOMAX PLUS), with concurrent video recording to enhance productivity and reduce errors (Bhagabati et al., 2012). The number of cycles to failure (NCF) was described by the equation: "NCF = Speed (RPM) X Time (T) to fracture in minutes." The study's armamentarium is depicted in Fig(1)

**A**

**B**

**C**

**Fig (1): The armamentarium used in this study**

 After being removed from the solutions, each file was rinsed with bi-distilled water to counteract the effects of NaOCl, dried, given an ID number, and stored in glass vials.

 Then, using a mechanical device created expressly for the job and capable of simulating the conditions of an instrument encased in a curved canal, instruments from all three groups and each brand were put through cyclic fatigue testing. (Grande et al. 2006, Plotino et al. 2009).

 The apparatus was connected to the same dynamic immersion programs and motor set. This made it possible for the endodontic instruments to freely reciprocate and maintain constant pressure inside a stainless steel artificial channel. To make the artificial canal, the instrument's dimensions and taper were reproduced. When the file made contact with the canal walls in the simulated canal, a special high-flow synthetic lubricant (Super Oil; Singer Co Ltd, Elizabethport, NJ, USA) was sprayed into the canal to lessen friction. The time to fracture (TtF) for each instrument, measured in seconds from the start of the test until the point of breakage, was recorded and registered using a chronometer to the nearest whole number. The TtF was the dependent variable, whereas the type of files used and the immersion conditions were independent factors.

**Statistical Analysis:**

           The Statistical Analysis System- SAS (2018) application was used to determine the effects of various factors on the research parameters. The least significant difference (LSD) test was used in this investigation to compare the means in a significant manner (ANOVA).

**Results**

 Descriptive statistics of TtF for the each file are summarized in Table (1 and 2).

**Table 1: Effect of  type of File and chemical materials in Length of**

**Chemical materials**

**Mean ± SE of Length of fracture**

**LSD**

**P-value**

**Edge file**

**Fanta file**

**Glycerin**

**3.02 ±0.17**

**C     b**

**3.65 ±0.16**

**A     a**

0.490 *

**0.0136**

**EDTA**

**4.74 ±0.11**

**A     a**

**3.45 ±0.09**

**A     b**

0.304 **

**0.0001**

**Sodium hypo-chloride**

**3.73 ±0.12**

**B     a**

**3.28 ±0.16**

**A     b**

0.421 *

**0.0371**

**LSD**

0.397 **

**0.413 NS**

**---**

**P-value**

**0.0001**

**0.199**

Means with different big letters in the same column and small letters in the same row are significantly different ,  * (P≤0.05),  ** (P≤0.01).

**Table 2: Effect of  type of File and chemical materials in Time**

**Chemical materials**

**Mean ± SE of Time (sec.)**

**LSD**

**P-value**

**Edge file**

**Fanta file**

**Glycerin**

**8.85 ±0.32**

**A     a**

**4.48 ±0.14**

**A     b**

0.725 **

**0.0001**

**EDTA**

**5.61 ±0.29**

**B     a**

**3.70 ±0.22**

**B     b**

0.764 **

**0.0001**

**Sodium hypo-chloride**

**5.45 ±0.09**

**B     a**

**3.63 ±0.24**

**B     b**

0.543 **

**0.0001**

**LSD**

0.741 **

0.598 **

**---**

**P-value**

**0.0001**

**0.010**

Means with different big letters in the same column and small letters in the same row are significantly different ,  ** (P≤0.01).

 In the table (1), we notice different significant differences in the effect of three materials on the edge file, which appeared a high significant differences for the material EDTA, then NaOH, then Glycerin in respectively, while there are no significant differences of the chemical materials on the Fanta file. While In the table (2), we notice very high significant differences between the effect of glycerin and the other two types of chemical materials, while appeared no significant differences between the two chemical materials when compared with each other on the two types of files.

 Note:- Averages that carry different letters in table (1 and 2) are significantly different, and averages that carry similar letters do not differ significantly. The highest average takes the letter a and so on downwards. If you find an average that takes two letters like ab, this is no different neither from the average that carries a nor from the average that carries b. as appeared in figure (2 and 3)

Link to Figure 2

**Figure (2) the effect of types of file and chemical material on length of fractures**

Link to Figure 3

**Figure (3) the effect of types of file and chemical material on time**

**Discussion**

 Despite considerable advancements in NiTi instrument design and technology, the persistence of NiTi instrument failure during root canal therapy remains a significant issue. These instruments are prone to fracture without apparent symptoms of permanent distortion. Even with the development of new endodontic instrument generations employing various manufacturing processes, the possibility of instrument fracture still exists.

 Every file system must undergo disinfection for infection control before reuse. While performing their functions, instruments are exposed to commonly used irrigants such as NaOCl, which fills the canals during instrumentation. Although various research efforts have assessed the cyclic fatigue resistance of different file systems, the effects of autoclaving and exposure during operation on various file systems' properties may vary. Consequently, this study contrasts multiple file systems, utilizing diverse manufacturing technologies, for cyclic fatigue resistance after exposure to NaOCl, glycine, and EDTA.

 The study aims to evaluate how irrigation affects the resistance to cyclic fatigue of two rotary endodontic instruments. Chemically active irrigation solutions are used during the clinical shaping of curved root canals. Surface interactions between the file and canal walls during this process could lead to corrosion or surface roughness, potentially resulting in fissures and cyclic fatigue of the file (Hasegawa et al., 2014).

 Various researchers have employed different methods, including pre-immersion of NiTi files in an irrigation solution or testing files for cyclic fatigue in synthetic oil, to evaluate the fatigue failure process (Pedulla et al., 2014).

 The interaction between the file and the irrigation solutions while the file is rotating in the irrigation solution is not taken into account by this design. In additional studies, the files were evaluated in an irrigation solution bath (Elnaghy and Elsaka, 2017). However, the relationship between the file and the canal walls was not taken into account.

 The files were tested while rotating in a curved glass tube filled with the irrigation fluid as part of the current study's specially created apparatus, which duplicates the clinical settings. To prevent galvanic corrosion from happening during testing in metal to or in contact with metal pins, as was stated in the previous research, a heat-resistant curved glass tube was employed (Shen et al., 2012). Glass tubes, which are not found in natural teeth, may have several drawbacks that come from chemical reactions with various irrigation solutions and should not be extrapolated clinically.

 Theoretically, employing aqueous media will allow the heat generated by friction to evaporate and gradually lengthen the fatigue lifetime of NiTi files. However, the current study's findings, which are in agreement with some reports (Shen et al., 2012; Hasegawa et al., 2014; Pedulla et al., 2011) and disagreement with others, show that the NCF of instruments tested in dry conditions without lubricant or coolant was significantly different from those tested in EDTA, Glycine, and NaOCl groups (Berutti et al., 2006; Elnaghy and Elsaka, 2017; Peters et al., 2007) These contradictory results could be the consequence of one of two factors.

 The first is that these aqueous solutions may hurtan an adverse effect on cyclic fatigue due to their propensity to produce corrosion and the roughness of the NiTi alloy surface. As a result, the fatigue life did not improve. The second factor is that fatigue testing occurred in a location with minimal cyclic fatigue. Regarding the first factor, prior studies indicated that immersion in NaOCl and EDTA could result in file corrosion (Berutti et al., 2006; Peter et al., 2007).

 However, in these studies, the file and shank were fully submerged for a long period of time, which led to galvanic corrosion, which is clinically inapplicable. Recent studies, which concur with the present findings, have shown that immersion does not, on the other hand, promote corrosion (Shen et al., 2012; Pedulla et al., 2011) or exhibit any adverse impact on cyclic fatigue even with an increased surface roughness of the files.

 The second theory appears more believable. The NCF in the existing configuration was in the low-cyclic fatigue range (Cheung et al., 2007; Tobushi et al., 1997). This amount of time is insufficient to demonstrate how the various circumstances interact. When the number of cycles fell within the low-cyclic fatigue life, Shen et al. (2012) showed no difference in NCF between the NCF of various files in dry conditions and a bath of irrigation solutions including Glycine, EDTA, and NaOCl. In contrast to dry conditions, liquid media has shown improvement in cycle fatigue in long fatigue life. In the landmark study by Tobushi et al. (1997), the researchers discovered that in areas of mild tiredness, there was no difference between wet and dry environments.

 Similarly to this, Pedullà et al. (2014) demonstrated that submerging NiTi files in EDTA and NaOCl had no detrimental effects on the NCF. On the other hand, testing WaveOne Gold in the air revealed better fatigue resistanceto fatigue compared to aqueous media, according to Elnaghy and Elsaka (2017). The methodology, instrument design, and type of motion employed may all be to fault for the inconsistent outcomes.

 The degree of curvature is another element that could obscure the effect of irrigation solutions on cycle fatigue. It is understood that a file moving through a steep curve experiences a significant strain amplitude, hastening the failure process (Cheung et al., 2007). A 60° curvature was used in the existing configuration. As a result, there wasn't enough time to demonstrate how a different environment (a low-fatigue region) affected things.

 Hasegawa et al. (2014) revealed that when evaluated in a 60° curved environment, three separate files examined in various conditions had similar NCF results. When the files were examined under a 30° arc of curvature, water increased the NFC of the files, but a different NFC was recorded. So it stands to reason that aqueous solution performance would be greater with longer testing periods.

 It's interesting to note that two fracture sites in the NaOCl group spread toward the file's center after being launched from opposing cutting edges. This might be connected to surface roughness, which could serve as a site for crack propagation. Thus, many cracks might account for the group's lower cycle count. The short testing period is the study's main drawback. The impact of irrigation solutions in areas with high cyclic fatigue should be evaluated. Additionally, analyzing the frictional forces that each solution generates and how they might affect the NCF may be helpful.

**Conclusion**

 The comparison between the two instruments that were tested revealed that NaOCl, glycine, and EDTA, as chemical materials, exhibited noticeably different cyclic fatigue resistance for various lengths of fractures and timeframes. The cyclic fatigue of NiTi files may be influenced by the irrigation environment. Chemical materials enhanced the files' Number of Cycles to Failure (NCF). However, due to the brief testing period (in the low-cyclic fatigue region), all other settings did not show any differences. Since cyclic fatigue takes much longer to develop than shaping actual teeth, the results of this in vitro study should not be directly applied to clinical situations. Therefore, caution is essential when drawing clinical conclusions. Nevertheless, further research is necessary to fully understand the various variables that can impact an instrument's cyclic fatigue resistance, fracture modes, and novel apparatus designs that share more properties with root dentine. Additional research is, therefore, required to validate and support the results of the current study.
